# A Quantum-Chemical Bonding Database for Solid-State Materials

**DOI:** 10.1038/s41597-023-02477-5

**Published:** 2023-09-11

**Authors:** Aakash Ashok Naik, Christina Ertural, Nidal Dhamrait, Philipp Benner, Janine George

**Affiliations:** 1 Federal Institute for Materials Research and Testing, Department Materials Chemistry, Berlin, 12205 Germany; 2 Friedrich Schiller University Jena, Institute of Condensed Matter Theory and Solid-State Optics, Jena, 07743 Germany; 3 Federal Institute for Materials Research and Testing, eScience Group, Berlin, 12205 Germany

**Keywords:** Electronic structure, Density functional theory, Electronic properties and materials

## Abstract

An in-depth insight into the chemistry and nature of the individual chemical bonds is essential for understanding materials. Bonding analysis is thus expected to provide important features for large-scale data analysis and machine learning of material properties. Such chemical bonding information can be computed using the *LOBSTER* software package, which post-processes modern density functional theory data by projecting the plane wave-based wave functions onto an atomic orbital basis. With the help of a fully automatic workflow, the *VASP* and *LOBSTER* software packages are used to generate the data. We then perform bonding analyses on 1520 compounds (insulators and semiconductors) and provide the results as a database. The projected densities of states and bonding indicators are benchmarked on standard density-functional theory computations and available heuristics, respectively. Lastly, we illustrate the predictive power of bonding descriptors by constructing a machine learning model for phononic properties, which shows an increase in prediction accuracies by 27% (mean absolute errors) compared to a benchmark model differing only by not relying on any quantum-chemical bonding features.

## Background & Summary

Understanding the interactions between constituent atoms in crystalline materials paves the way for developing and tailoring novel solid-state materials with desired application-specific properties^1–4^. For instance, the ultra-low lattice thermal conductivity in thermoelectric materials is connected to strong antibonding interactions^5,6^. Bonding analysis aids in quantifying such interatomic interactions, and several theoretical frameworks exist. Popular and well-known approaches are the Atoms In Molecules (AIM) approach to derive electron density-based Bader charges^7^, or wave function-based concepts like the Mulliken population analysis^8^, from which Crystal Orbital Overlap Populations (COOP)^9^, Crystal Orbital Hamilton Populations (COHP)^10^, and the Crystal Orbital Bond Index (COBI)^11^ are derived.

Nowadays, many robust automation frameworks for simulation have become available^12–16^. These automation tools allow for high-throughput calculations on a scale of thousands of materials^17–19^. Reusing such large amounts of data as inputs for machine learning algorithms has enabled data-driven material science research for accelerated discovery of novel materials and gaining a better understanding between materials structure and properties^6,20^.

For solid-state materials, plane wave-based basis sets provide easy means to exploit periodicity and gain computational efficiency due to their delocalized nature when performing atomistic simulations via density functional theory (DFT). This computational efficiency comes at the cost of losing crucial atom-specific chemical bonding information. The Local-Orbital Basis Suite Towards Electronic-Structure Reconstruction (*LOBSTER*)^21–24^ software package can recover such bonding information by projecting plane-wave-based wave functions onto atomic orbitals. Since its first release, this program has been used extensively to study different materials classes (e.g, phase-change materials^25,26^, Li/Na ion battery^27,28^, low thermal conductivity materials^5,29–31^) and to uncover the diverse underlying atomistic phenomena in the respective bonding mechanisms^26,28,31^. Although high-throughput materials design and research studies with data have been conducted in a few cases^32–35^, no dedicated database exists to retrieve and reuse such data. Previous studies have clearly shown that bonding data computed with *LOBSTER* is of high value for the materials informatics community, and we provide an open-access database of bonding information here for the first time.

In this work, we perform bonding analysis for 1520 compounds using an automated workflow^36^ recently developed by some of us that combines Vienna Ab initio Simulation Package (*VASP*)^37–39^ DFT computations with *LOBSTER* calculations using Python tools like *pymatgen*^40^, *atomate*^14^, and *FireWorks*^41^. To generate summarized bonding information ready to be used for machine learning studies, we used the *LobsterPy*^36,42^ package that automatically analyzes *LOBSTER* COHP output files. We provide this summarized bonding information data as (lightweight) JSON files. We also distribute all relevant *LOBSTER* computation data validated and formatted using a *pydantic* schema, including all the settings and relevant output files.

In the following sections, we begin by briefly summarizing the computational details of the workflow employed to perform the computations. We then describe the method used to generate entries in the database and provide an overview of the structure of the database. Finally, we benchmark the quality of our results by comparing them with projected densities of states from a widely-used density-functional theory code and available heuristics for bond valences and coordination environments. Lastly, we demonstrate the influence of including quantum-chemical bonding data in a machine learning model for predicting phononic properties.

## Methods

### Structures

We included a total of 1520 crystalline materials in this work. The Materials Project (MP) database^43^ is used to retrieve all the structures. These materials belong to a previously published dataset of harmonic phonon properties including band structures and densities of states^19^. We selected this database as it consists only of semiconductors and insulators. For these materials, it is easier to choose a local basis set for the* LOBSTER* projection as they have clearly distinguished valence and conducting states separated by a band gap. We chose a minimal Slater-type orbital (STO) basis, as provided in *LOBSTER*, consisting only of occupied valence orbitals in the atomic ground state of each atom (as used in the projector-augmented wave method).

### Bonding indicators definitions

*LOBSTER* first projects the projector-augmented wave (PAW) wavefunctions obtained from DFT computations onto a local STO basis to quantify the interatomic interactions. Combining the coefficients of linear combinations of atomic orbitals (LCAO) generated from this projection with overlap, Hamiltonian, and density matrices, quantum-chemical bonding characteristics in materials are estimated. Here, we summarize the key quantities computed by *LOBSTER*, and the notations used follow the same convention as in refs. ^11,24^:

1 $${{\rm{pCOOP}}}_{\mu \nu }(E)={S}_{\mu \nu }\sum _{j,k}{w}_{k}Re\left({c}_{\mu ,jk}^{* }{c}_{\nu ,jk}\right)\cdot \delta \left({\varepsilon }_{j}(k)-E\right)$$

2 $${{\rm{pCOHP}}}_{\mu \nu }(E)={H}_{\mu \nu }\sum _{j,k}{w}_{k}Re\left({c}_{\mu ,jk}^{* }{c}_{\nu ,jk}\right)\cdot \delta \left({\varepsilon }_{j}(k)-E\right)$$

3 $${{\rm{COBI}}}_{\mu \nu }(E)={P}_{\mu \nu }\mathop{}\limits_{}\sum _{j,k}{w}_{k}Re({c}_{\mu ,jk}^{* }{c}_{\nu ,jk})\cdot \delta ({\varepsilon }_{j}(k)-E)$$

The overlap, Hamiltonian and density matrix between orbitals Φ_*μ*_ and Φ_*v*_ are represented by *S*_*μv*_, *H*_*μv*_ and *P*_*μv*_ respectively. *w*_*k*_ is the *k*-point weight, and ${c}_{\mu ,jk;\nu ,jk}$ are the coefficients of LCAOs. *Re* indicates the real part of the complex value. ${\varepsilon }_{j}(k)$ and *E* represent the energy eigenvalue of band *j* at *k* within the Brillouin zone and the general energy, respectively. The energy-integrated values (up to the Fermi level) of these quantities, namely ICOOP, ICOHP, and ICOBI, can be interpreted as the number of electrons in the bond, a measure of bond covalency (corresponding to covalent bond strength), and bond order, respectively.

*LOBSTER* also provides Mulliken and Löwdin atomic charges from the orbital-derived atomic gross populations (GP)^44^. The Madelung energy is derived using Mulliken or Löwdin atomic charges as input. Madelung energies represent the electrostatic part of the lattice energy and can be related to the stability of ionic crystal structures. For details about the mathematical formulation related to Madelung energies, Mulliken, and Löwdin atomic charges in *LOBSTER*, we refer the readers to ref. ^11^ and the literature referenced therein.

### Workflow and computational parameters

**Fig. 1:**
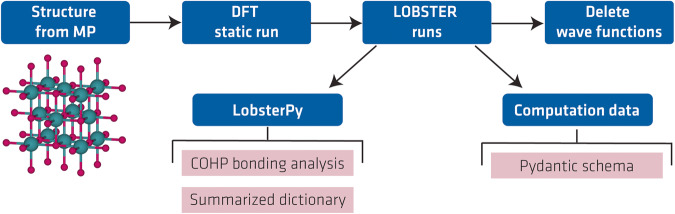
Workflow schematic for computations and data record generation.

To create the database, we used an automatic bonding analysis workflow^36^ developed recently by some of us. To start this workflow, one must provide the crystal structure as input. Based on the input structure, it performs the bonding analysis with the *LOBSTER*^24^ program by adding all necessary computational steps to the pipeline. To summarize, these steps involve (a) writing *VASP* input files with an appropriate number of bands (NBANDS) for a static DFT run, (b) a static DFT run, (c) writing input files for *LOBSTER* runs with all available atomic orbital basis functions for the projection of the wave function, (d) *LOBSTER* runs, (e) deleting (disk-space consuming) wave function (“WAVECAR”) files. Figure 1 shows the schematic sequence of our workflow.

Within this workflow, the DFT computations were performed using the generalized gradient approximation (GGA) functional as parameterized by Perdew, Burke, and Ernzerhof (PBE)^45,46^ within the PAW framework^47,48^. We employ a grid density of 6000 *k*-points per reciprocal atom and set NEDOS (number of energy points on which the density of states is evaluated) to 10000 points. The electronic structure’s convergence criterion is set to 10^−6 ^eV, and the plane-wave energy cutoff is set to the standard value of 520 eV, as implemented in the original workflow. The Brillouin zone is integrated using the tetrahedron method with Blöchl correction^49^ (i.e., ISMEAR = −5). All computations were performed including spin polarization. For COHP computations using *LOBSTER*, we use the entire energy range of *VASP* static runs, and COHP steps are set equal to the NEDOS (i.e., 10000 steps) set for *VASP* static run. We stress that the number of COHP steps does not influence the ICOHP values provided in the “ICOHPLIST.lobster” files; only the energy ranges and increments in the “DOSCAR.lobster” and “CO**CAR.lobster” are influenced. We increased the number of points for the DOS computation to be able to benchmark the *LOBSTER* projected DOS with the help of the *VASP* projected DOS. As both *LOBSTER* and *VASP* DOS were computed in the same workflow, the *VASP* DOS was also computed without symmetry (ISYM = 0), which is now also the recommended setting for *VASP* projected DOS for the *VASP* version that we used^50^. With this high number of points in the DOS and COHP computations, the bonding and anti-bonding percentage values from our automatic analysis of output files additionally also pose a very good estimate of bonding and anti-bonding contribution in bonds as we rely on a numerical integration in *LobsterPy*. All the unprocessed computational data is available (refs. ^51–58^). The code for starting the workflows is also provided for reproducibility.

### Generating data records

We provide data records in two forms. The smaller data record consists of summarized bonding information that is very lightweight and can be quickly assessed in seconds to retrieve and examine relevant bonds. The other, larger data record consists of all the *LOBSTER* computational data.

To generate the smaller data records including summarized bonding information (*LOBSTER* lightweight data), we used the “CondensedBondingAnalysis” schema implemented as part of the *atomate2*^59^ *LOBSTER* workflow. This schema automatically analyzes the *LOBSTER* output files in the “cation-anion” and “all” bond modes using the *LobsterPy*^36,42^ package. In cases without ions in the structure, only data from the analysis of all bonds are available. When the “cation-anion” mode is used, the automatic analysis detects cations and anions based on the Mulliken charges, and only “cation-anion” bonds are included in the analysis. Then, the strongest cation-anion bond is determined based on the Integrated Crystal Orbital Hamilton Populations (ICOHPs). To determine coordination environments and to perform automatic plots, only bonds with a strength of at least 10% of the strongest bond are considered. If the “all” mode is used, the other bonds are also included in the analysis. The schema also identifies the strongest bonds and corresponding bond lengths based on ICOHP, ICOOP, and ICOBI data for the relevant bond pairs as per *LobsterPy* bonding analysis. Additionally, we include Madelung energies and atomic charges based on Mulliken and Löwdin population analysis methods. Lastly, a summary of technical validation results, which consists of charge spillings, band overlaps analysis, density of states, and charge comparisons, is included, providing an overview of data quality. A larger data record (Computational data) with all the important *LOBSTER* computation data is generated using the “LobsterTaskDocument”, which is a *pydantic* schema again implemented as part of the *atomate2* *LOBSTER* workflow. This schema uses *LOBSTER* parsers implemented in the *pymatgen* package to read the *LOBSTER* files and store the information necessary to recreate the Python objects in the form of a Python dictionary. It also includes the *LobsterPy* data from smaller summarized bonding information data records. A code to generate and read these JSON files is also provided in the code repository for this publication. This allows easy means to reuse or access the data.

## Data Records

### LOBSTER lightweight data file format

The data is stored in JSON format (ref. ^60^). The files are named with the the Materials Project ID of the compound. Each JSON file includes summarized bonding information. Table 1 summarizes the root keys to access data from the JSON file. Table 2, explains the data inside the “all_bonds” and “cation_anion_bonds” keys. Tables 3, 4 explain the data found in the “lobsterpy_data” and “calc_quality_summary” keys of Tables 1, 2, respectively.

**Table 1:** Top level keys of the *LOBSTER* lightweight JSON files.

Root keys	Datatype	Description
all_bonds	dict	Summarized relevant bonds data (See Table 2 for details).
cation_anion_bonds	dict	Summarized relevant cation-anion bonds data (See Table 2 for details).
madelung_energies	dict	Total electrostatic energy for the structure as calculated from the Mulliken and Löwdin charges.
charges	dict	Atomic charges with Mulliken and Löwdin population analysis methods as keys. Each key’s corresponding list follows the order of sites in the crystal structure.
calc_quality_summary	dict	Dict summarizing results of technical validation tests such as charge spillings, band overlaps, DOS and charge comparisons. (See Table 3 for details).

**Table 2:** Keys corresponding to “all_bonds” and “cation_anion_bonds” in the *LOBSTER* lightweight data JSON file.

Root keys	Datatype	Description
lobsterpy_data	dict	Condensed bonding analysis data from *LobsterPy* (See Table 4 for details).
lobsterpy_text	string	Contains *LobsterPy* automatic analysis summary text.
sb_icobi	dict	Dict with the strongest ICOBI bonds.
sb_icohp	dict	Dict with the strongest ICOHP bonds.
sb_icoop	dict	Dict with the strongest ICOOP bonds.

**Table 3:** Keys corresponding to “calc_quality_summary” in the *LOBSTER* lightweight data JSON file.

Root keys	Datatype	Description
minimal_basis	bool	Bool indicating if the *LOBSTER* calculation used a minimal basis for projection
charge_spilling	dict	Contains the absolute charge spilling value
band_overlaps	dict	Dict summarizing important information from the “bandOverlaps.lobster” file to evaluate the quality of the projection, namely whether the file is generated during projection (i.e., larger deviations exist), the maximum deviation observed, percent of *k*-points above the threshold set in *pymatgen* parser (during data generation the value was set to 0.1)
dos_comparisons	dict	Dict with Tanimoto index values obtained from comparing *VASP* and *LOBSTER* projected DOS fingerprints

**Table 4:** Keys corresponding to “lobsterpy_data” in the *LOBSTER* lightweight data JSON file.

Root keys	Datatype	Description
formula	string	Chemical formula of the compound.
max_considered_bond_length	float	Maximum bond length that has been considered in the analysis.
limit_icohp	float array	Minimum and maximum ICOHP that has been considered in the analysis.
number_of_considered_ions	int	Number of ions that has been detected.
sites	string	Site index of the sites in the crystal structure for which bonds have been detected (nested dict that describes the bond and its co-ordination environment as determined based on the ICOHP values).
type_charges	string	Whether the Mulliken or the Lödin charges have been used for the bonding analysis.
cuttoff_icohp	float	ICOHP cutoff value set for bonding analysis.
summed_spins	bool	Indicates if spins are summed.
start	int	Sets the energy for evaluation of bonding and anti-bonding percentages based on COHP.
cohp_plot_data	dict	Relevant bond labels as keys and corresponding cohp objects to plot COHP curves from automatic analysis.
which_bonds	string	Indicates the mode of automatic bonding analysis run (“cation_anion” or “all”).
final_dict_bonds	dict	Includes relevant bond labels, ICOHP mean values and indicates if anti-bonding states below the Fermi level exist.
final_dict_ions	dict	Includes all different coordination environments and counts them.
run_time	float	Time needed in secs to run the automatic bonding analysis.

### Computational data file format

The data is stored in JSON format (refs. ^60,61^). The files are named as per the Materials Project ID of the compound. Each JSON file includes all the *LOBSTER* output files parsed and stored in the form of a Python dictionary. It also includes the summarized bonding analysis based on ICOHP values and contains the same information as explained in Table 2. Table 5 summarizes root keys to access data from the JSON file.

**Table 5:** Top level keys of the computational data JSON files.

Root Keys	Data type	Description
structure	dict	Dict representation of the *pymatgen* “Structure” object used for the *LOBSTER* calculation.
charges	dict	Atomic charges dict from *LOBSTER* based on the Mulliken and Löwdin charge analysis.
lobsterin	dict	*LOBSTER* calculation inputs.
lobsterout	dict	Information on *LOBSTER* calculation output.
lobsterpy_data	dict	Summarized bonding analysis data from *LobsterPy* (all bonds mode). It also includes “Cohp” objects to plot the COHP curves from the automatic analysis.
lobsterpy_text	dict	*LobsterPy* automatic analysis summary text (all bonds mode).
strongest_bonds_icohp	dict	Describes the strongest ICOHP bonds.
strongest_bonds_icoop	dict	Describes the strongest ICOOP bonds.
strongest_bonds_icobi	dict	Describes the strongest ICOBI bonds.
lobsterpy_data_cation_anion	dict	Summarized bonding analysis data from *LobsterPy* (cation-anion bonds mode). It also includes “Cohp” objects to plot the COHP curves from the automatic analysis.
lobsterpy_text_cation_anion	dict	*LobsterPy* automatic analysis summary text (cation-anion bonds mode).
strongest_bonds_icohp_cation_anion	dict	Describes the strongest cation-anion ICOHP bonds.
strongest_bonds_icoop_cation_anion	dict	Describes the strongest cation-anion ICOOP bonds.
strongest_bonds_icobi_cation_anion	dict	Describes the strongest cation-anion ICOBI bonds.
cohp_data	dict	Dict representation of *pymatgen* “CompleteCohp” object including data to plot COHP curves.
coop_data	dict	Dict representation of *pymatgen* “CompleteCohp” object including data to plot COOP curves.
cobi_data	dict	Dict representation of *pymatgen* “CompleteCohp” object including data to plot COBI curves.
dos	dict	Dict representation of *pymatgen* “LobsterCompleteDos” object including the “DOSCAR.lobster” data.
lso_dos	dict	Dict representation of *pymatgen* “LobsterCompleteDos” object including the “DOSCAR.LSO.lobster” data.
madelung_energies	dict	Consists of the Madelung energies of the structure derived from the Mulliken and Löwdin charges.
site_potentials	dict	Site potentials dict based on Mulliken and Löwdin charges.
gross_populations	dict	Gross populations dict based on Mulliken and Löwdin charges with each site as a key and the gross population as a value.
band_overlaps	dict	Band overlaps matrices for each *k*-point as extracted from the “bandOverlaps.lobster” file, if it is generated during the LOBSTER run.

## Technical Validation

### Projection quality

**Fig. 2:**
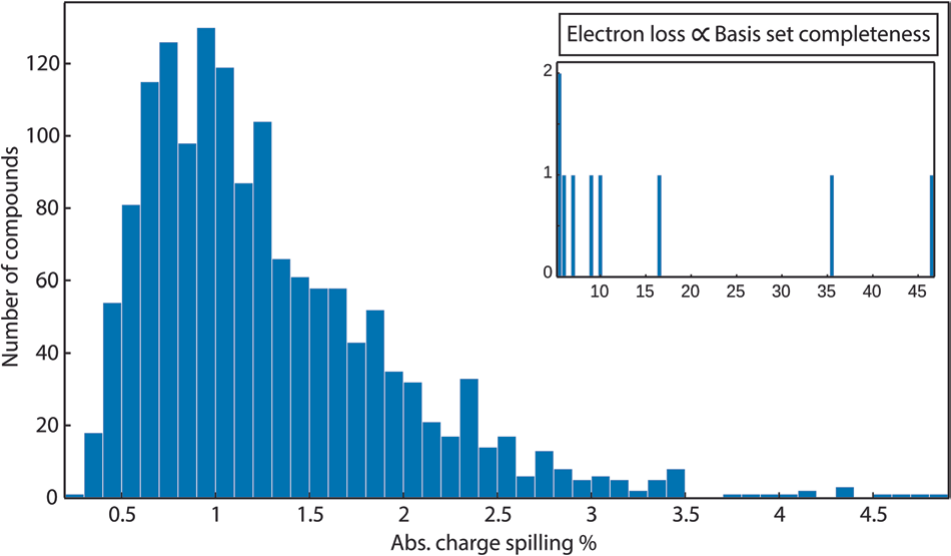
Distribution of the absolute charge spilling from the *LOBSTER* computations for the entire data set (compounds with a spilling > 5% are shown in the inset). Possible reasons for the nine outliers are discussed in the text.

The absolute charge spilling reported at the end of the *LOBSTER* calculations indicates the quality of the projection corresponding to the loss of charge density that occurs when projecting the original PAW functions onto the local basis. Ideally, when the provided local basis set is complete (i.e., properly reproducing the PAW-based Hilbert space and representing the chemistry of the compound in question), the charge spilling value approaches zero, indicating the reliability of the results. Figure 2 below shows the distribution of the charge spilling for our data set. Approximately 99% of compounds have charge spilling of <5%.

Only a very few compounds show a charge spilling of >5%, possibly due to the limited basis function availability in *LOBSTER*. The nine compounds showing an absolute charge spilling >5% are BaO_2_ (mp-1105), SiC (mp-11713), Be_2_C (mp-1569), Li_4_NCl (mp-29149), CsBiO_2_ (mp-29506), Cs_2_O (mp-7988), KYO_2_ (mp-8409), Rb_2_PtSe_2_ (mp-8622) and SrHfN_2_ (mp-9383), with spillings ranging between 5.5 and almost 50% (see inset in Fig. 2). The most extreme case is BaO_2_ with an absolute charge spilling of 46.7%. As the absolute charge spilling determines how well the *VASP* and *LOBSTER* wave function match each other for occupied orbitals, two possible reasons for this outlier that are interconnected are coming into consideration: in this particular case, the electronic structure is very sensitive to small changes in the structure and/or the provided basis functions are not sufficient for a proper projection. An additional optimization of the MP structure changes the wave function so that the projection without 5d orbitals arrives at an acceptable absolute charge spilling of 3.91%. Furthermore, with an experimental version of *LOBSTER*^62^, that allows to include arbitrary orbitals into the projection, adding the La 5d orbital to Ba, as the *VASP* POTCAR suggests a 5d occupation of 0.010, the absolute charge spilling drops to 1.40% without further structural optimization. Unfortunately, *LOBSTER* currently does not provide atomic orbitals for 5d orbitals of Ba.

*LOBSTER* also generates a “bandOverlaps.lobster” file as another measure of projection quality for the cases when the projected wave function is not orthonormalized with an accuracy of 10^−5^ for every *k*-point. This file contains the band overlap matrices of the projected bands for each *k*-point that allows analyzing how well the projected wave function is orthonormalized, and the maximal deviation from the identity matrix is indicated as well. In ideal cases, the deviation should approach zero. Achieving this numerically is almost impossible. Thus, it does not generally indicate a critical error; nevertheless, we analyzed the data from these files for our complete dataset. We set the off-diagonal matrix element deviation threshold for this analysis to 0.1. We then evaluated the percentage of *k*-points for each compound for which the deviation is larger than the deviation threshold. It is found that approximately 7% of the compounds in the database show 5% or more *k*-points above this threshold. We have included these compounds in the rest of the analysis and the database, as the other benchmarked results still show sufficient agreement. However, the bonding information from these compounds should be used with caution.

Overall, these results demonstrate that the local basis used for our computations correctly represents the material’s chemistry for the majority of compounds. The *LOBSTER* projection mismatch (abs. charge spilling >5%) also helps to figure out problematic basis set functions as discussed in the case of BaO_2_.

### Projected density of states (PDOS) benchmarking

As *LOBSTER* quantifies the interatomic interactions by projecting the PAW wavefunctions from DFT computations (in our case: *VASP*) onto a provided local orbital basis, it also generates PDOS that is independent of the PDOS generated by *VASP*. But unlike the *LOBSTER* projection, the *VASP* projection typically loses more electron density when using standard Wigner-Seitz radii. Nevertheless, we will use the VASP projection data for benchmarking as this data is commonly used in the field, and automation are available. We will, however, not compare the absolute projected density of state values for this reason. A common way to compare the density of states relies on visual inspection of relevant features. However, with thousands of PDOS plots, performing a visual inspection is not feasible. To numerically compare the PDOS from *VASP* and *LOBSTER*, we have chosen several methods that do not rely on the absolute values but instead on features of the PDOS that are relevant for understanding the electronic structure of a material. First, we compute moments of the PDOS from *VASP* and *LOBSTER*. These moments, in principle, provide an estimate of the shape of the PDOS in the selected energy range. Namely, we compare here the band center (1^*st*^ moment)^63^, bandwidth (the $\sqrt{{2}^{nd}}$ moment), band skewness (the 3^*rd*^ standardized moment), and kurtosis (the 4^*th*^ standardized moment) of the band directly below the Fermi level (*E*_*F*_). These features provide an overview of the numerical similarity of DOS and are easy to evaluate using existing methods implemented in the “electronic_structure.dos” module in *pymatgen*^40,64^. It must be noted that we compare the Löwdin symmetric orthonormalized (LSO) DOS obtained from *LOBSTER*, which recovers the entire Hilbert space and ensures that no electron density is lost, to the *VASP* projected DOS.

**Fig. 3:**
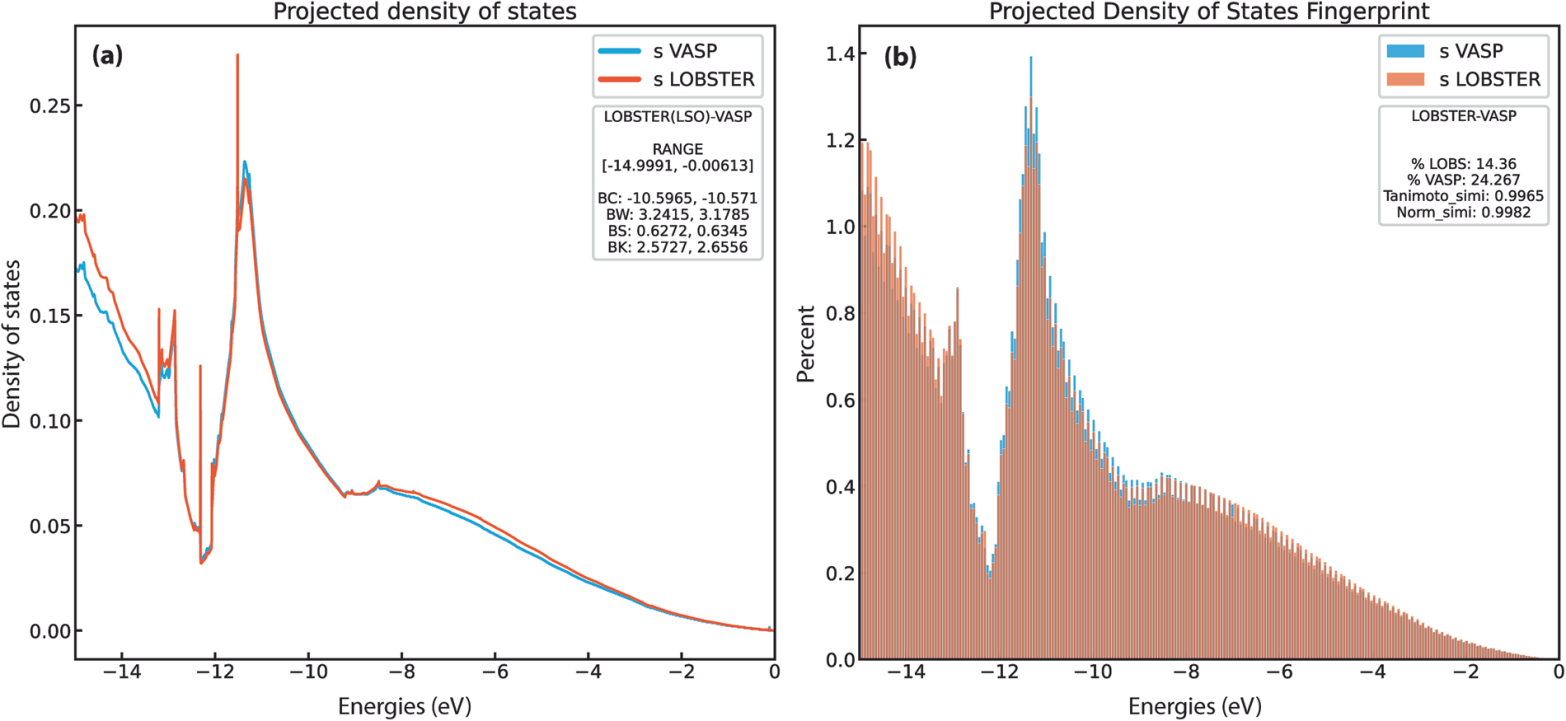
(**a**) Band features and (**b**) fingerprint exemplar plots for PDOS from *LOBSTER* and *VASP* runs for diamond (mp-66). In subfigure (**a**), BC, BW, BS, and BK denote band center, width, skewness, and kurtosis, respectively. The percentages of orbital contribution in the chosen energy range are shown in subfigure (**b**) as % LOBS and %VASP. The Tanimoto index and the normalized vector dot product, respectively, are denoted by the Tanimoto_simi and Norm_simi.

**Fig. 4:**
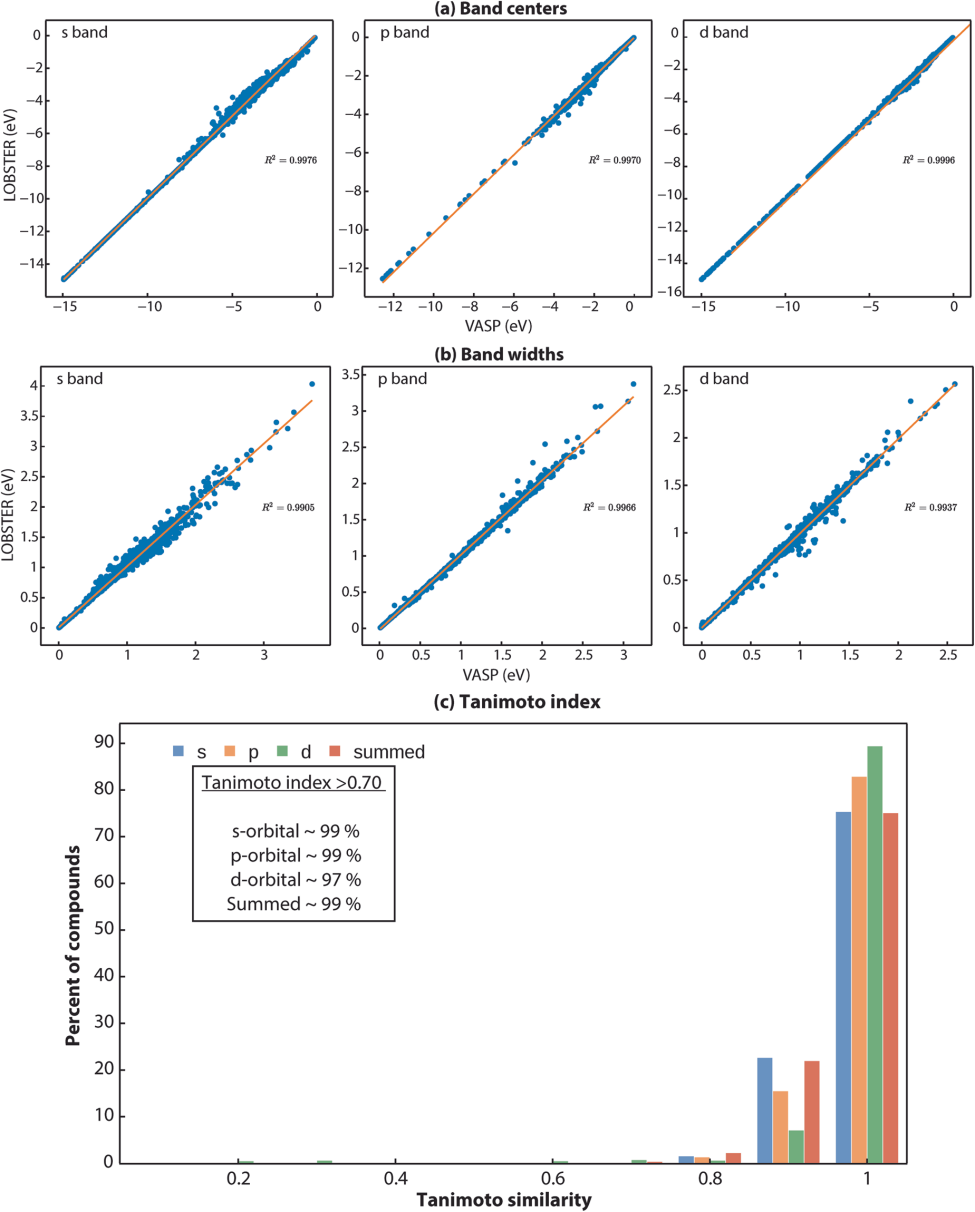
(**a**) Band centers and (**b**) band width comparison of projected DOS (s, p and d bands) for the first energy range without PDOS values close or equal to zero below the Fermi level (*E*_*F*_) obtained from *LOBSTER* and *VASP* runs. Both figures show that projected DOS from *LOBSTER* runs agree very well with our reference *VASP* data. (**c**) Histogram of Tanimoto index (*S*_*A, B*_) computed between *VASP* and *LOBSTER* PDOS (Summed denotes the sum of all individual PDOS).

**Fig. 5:**
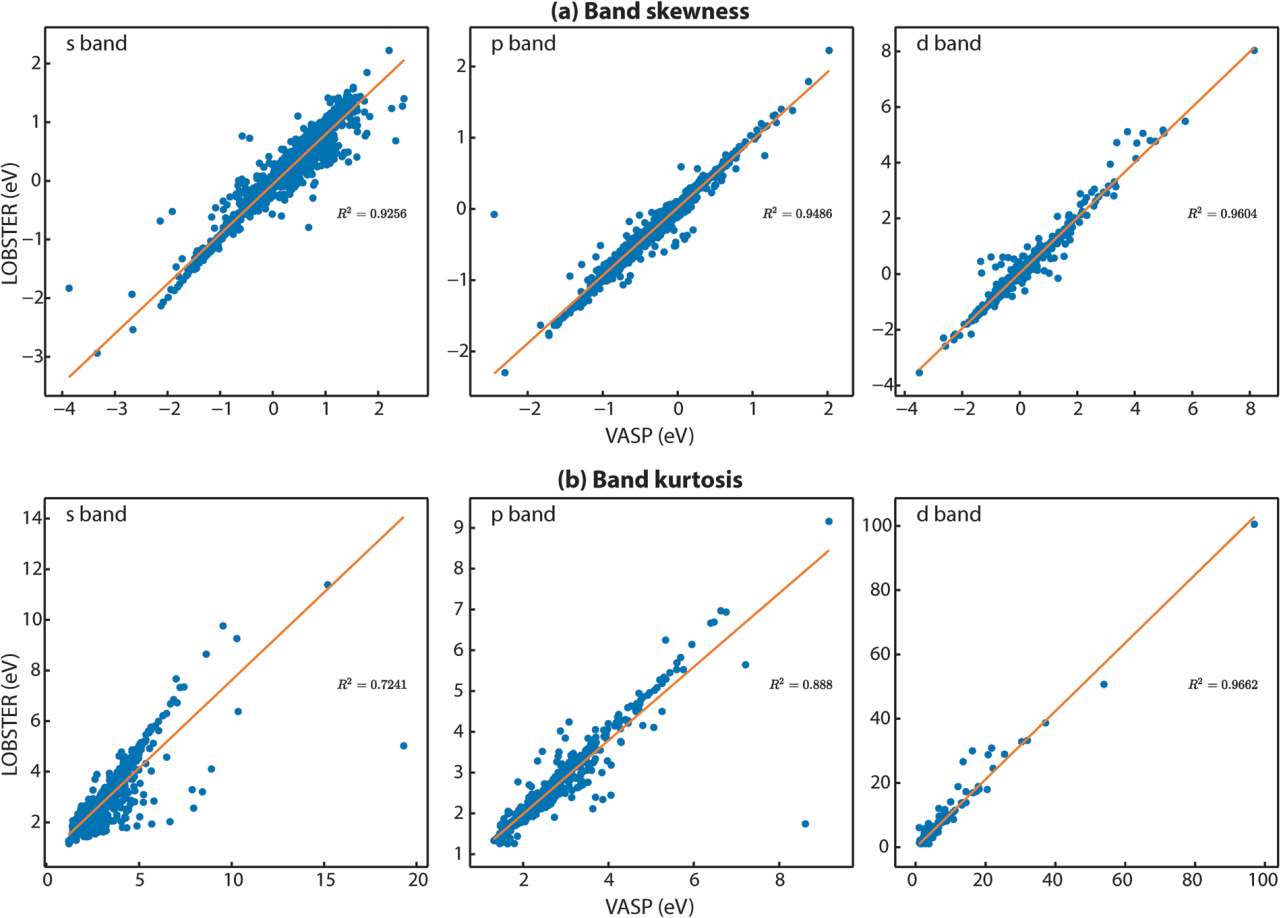
(**a**) Band skewness and (**b**) band kurtosis comparison of projected DOS (s, p and d bands) for first non-zero energy range below the Fermi level (*E*_*F*_) obtained from *LOBSTER* and *VASP* runs. Both figures show that the projected DOS from *LOBSTER*runs are in reasonable agreement with our reference *VASP* data.

**Fig. 6:**
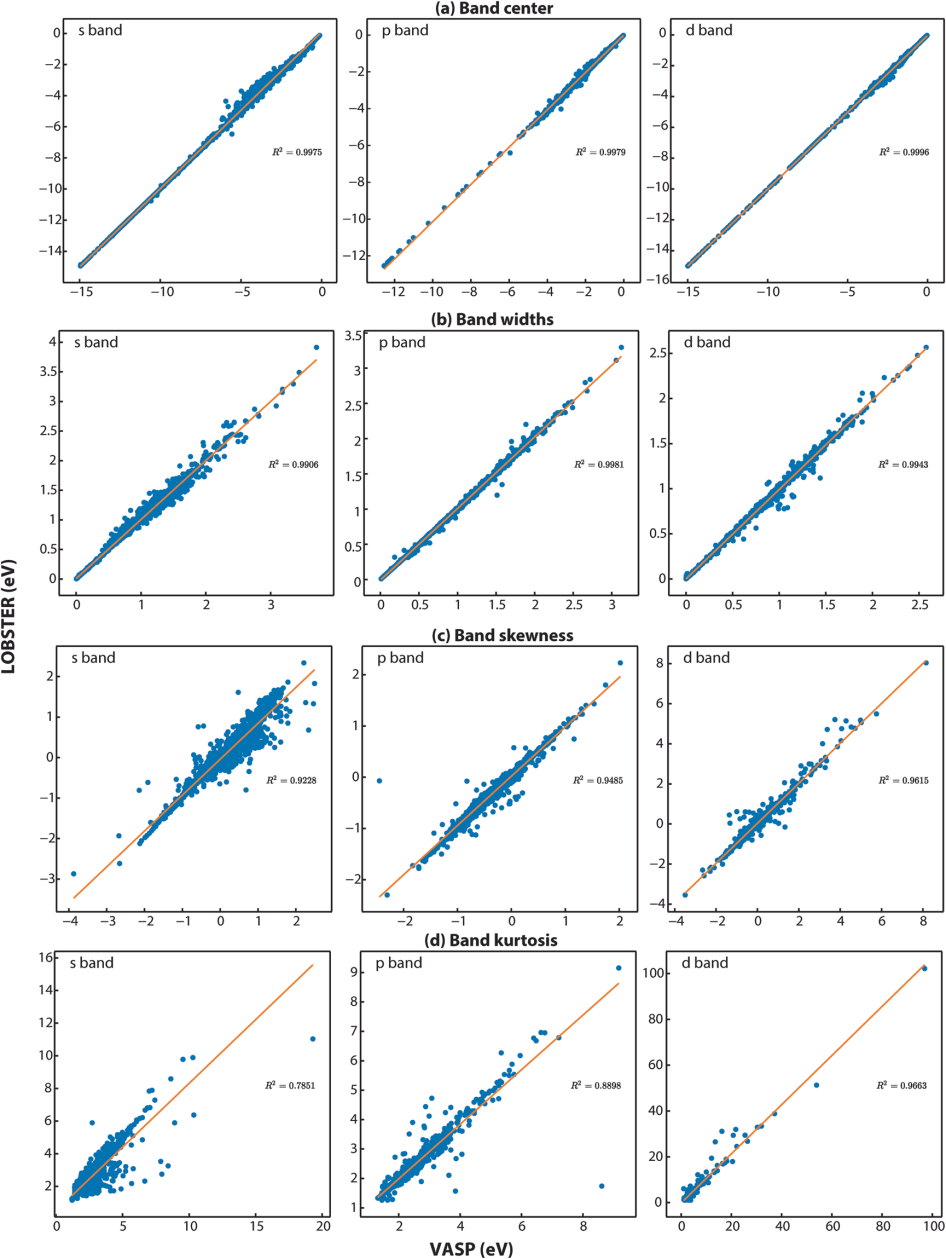
(**a**) Band center and (**b**) width (**c**) skewness and (**d**) kurtosis comparison of projected DOS (s, p and d bands) for first non-zero energy range below the Fermi level (*E*_*F*_) obtained from *LOBSTER* (non LSO) and *VASP* runs.

To compute the PDOS features, we first extract all energy ranges below *E*_*F*_ in which the PDOS is not equal or close to zero. Next, we use the energy range just below *E*_*F*_, where a non-zero PDOS is detected, to evaluate the PDOS moment features. To ensure that the obtained energy ranges significantly contribute to the overall band, we set a threshold of 0.5 electrons for the band feature comparisons. Figure 3a provides exemplar plots for comparing the PDOS. As evident from the band features, a sufficient agreement exists in this particular case (diamond, mp-66) between *VASP* and LOBSTER data. In Fig. 4a,b, we compare projected DOS for s, p, and d band centers and band widths obtained from our *VASP* and *LOBSTER* runs for the whole data set, respectively. A very good agreement is visible for most compounds. In Fig. 5, we report comparisons of PDOS features, namely band skewness and kurtosis. A comparison of the non-LSO DOS is also shown in the in Fig. 6.

Another way to assess the similarity between PDOS is to compute Tanimoto coefficients. Earlier studies have demonstrated that such a measure is not only suitable to compute the similarity between molecules^65^ but is also a reliable way to compare DOS of materials^66^. The formula to compute the Tanimoto coefficient is as follows:

4 $${S}_{A,B}=\frac{A\cdot B}{| | A| {| }^{2}+| | B| {| }^{2}-A\cdot B}$$

The Tanimoto coefficient (*S*_*A,B*_) can be interpreted as the ratio of the dot product of the two vectors A and B to the sum of their magnitudes and the dissimilarity between them.

**Fig. 7:**
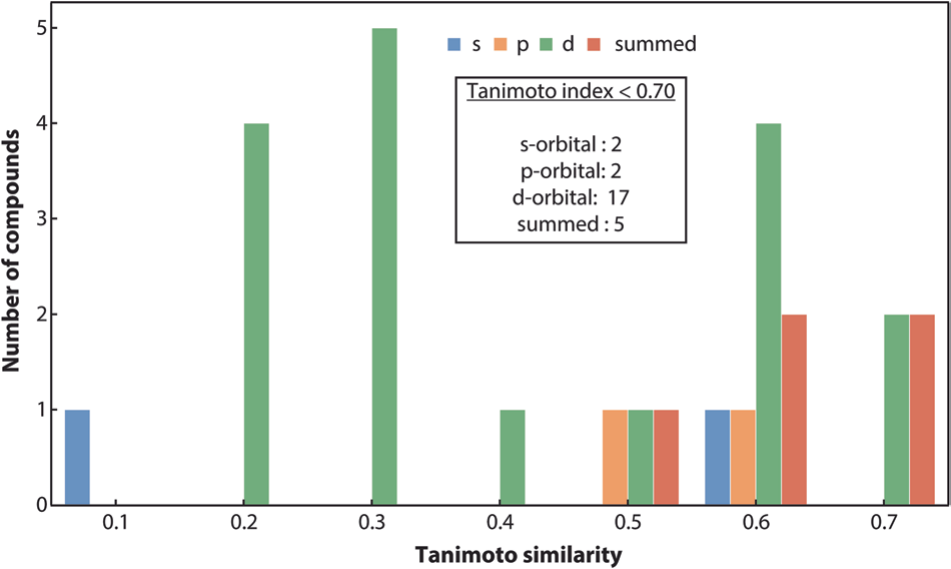
Histogram of Tanimoto indices ( < 0.70) computed between *VASP* and *LOBSTER outputs*.

We adapted the “materials_fp” module of the FHI-vibes^67,68^ Python package to evaluate the similarity between the PDOS of the *VASP* and the *LOBSTER* program. The adapted code has been incorporated in the *pymatgen* package and has been publicly available since v2023.1.9. Here, we first discretize PDOS from *VASP* and *LOBSTER* in 256 bins and normalize it before computing the *S*_*A, B*_ for the energy range of −15 to 0 eV (energies are shifted relative to the Fermi energy) for all the compounds. Again, for diamond (mp-66) in Fig. 3b,we show the binning of the PDOS and the corresponding Tanimoto similarity, indicating very good agreement between *VASP* and *LOBSTER* data. Compounds, where the number of valence electrons obtained by integrating summed PDOS of VASP exceeded the actual valence electrons based on the POTCAR, are excluded from the analysis, as this indicates a poor projection. Again, we only compare PDOS if they significantly contribute to the density of states in the selected energy range. We have set this threshold to 5% of the sum of the projected DOS. Figure 4c shows the distribution of evaluated *S*_*A, B*_ for the subset of our dataset. We can see that, for most compounds, *S*_*A, B*_ lies in the range of 0.75 to 1. Approximately 99% of compounds have a similarity index of more than 0.70. Only a few cases exist where *S*_*A, B*_ is less than 0.70, as shown in Fig. 7. Disagreements are observed in cases where unusual sharp peaks occur in the projection or some low-lying states are missing in the *VASP* or *LOBSTER* projections. Overall our results demonstrate that the basic features of the PDOS from *VASP* and *LOBSTER* agree very well. Therefore, we can conclude that the *LOBSTER* projection was performed reliably and that we can compute bonding properties such as COHPs and COBIs of high quality based on this projection. We also provide an interactive dash app to explore these computed PDOS features visually for convenience (10.5281/zenodo.7795903).

### Further quality markers: Atomic charges and coordination environments

**Fig. 8:**
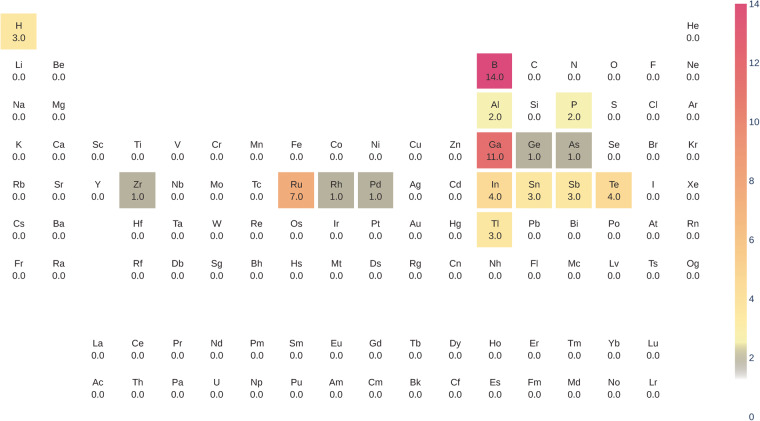
Elements for which cations and anions assignment classification differs between *LOBSTER* and the BVA methods depicted in the form of a heatmap. The heatmap was plotted with pymatviz^96^.

**Fig. 9:**
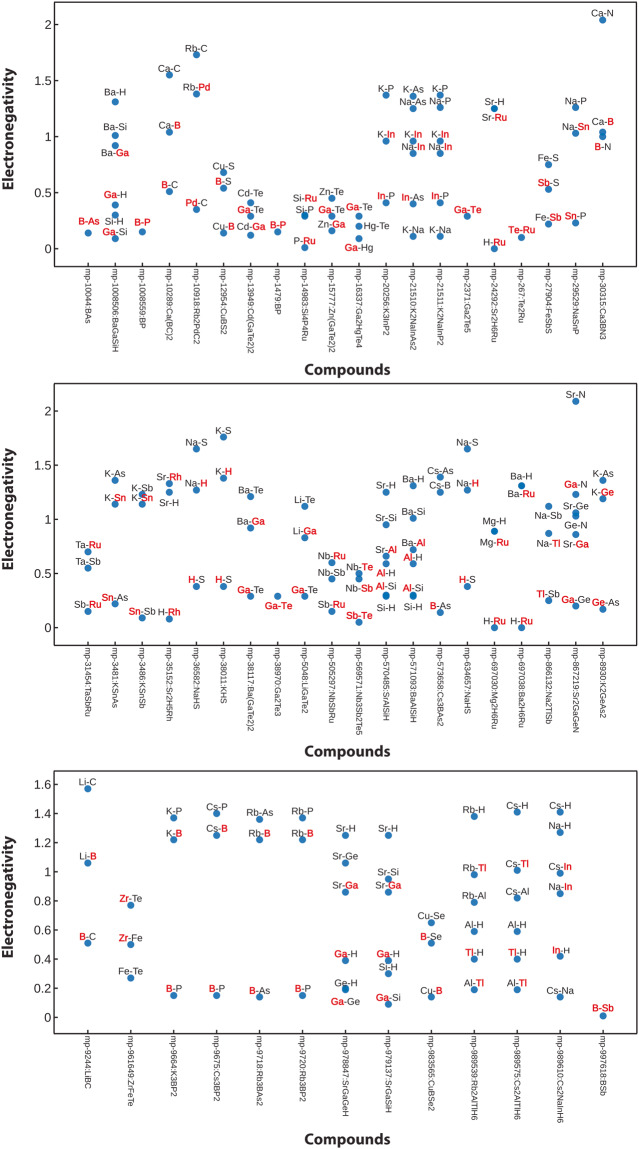
Electronegativity differences scatter plot for the compounds for which the assignment of cations and anions from *LOBSTER* and the BVA method differs. (Text annotations in RED depict the elements where cation-anion classification disagreements are observed).

While Mulliken and Löwdin charges from *LOBSTER* are derived using the LCAO coefficients and arrive at non-integer values^44^, the bond valence analysis (BVA)^69^ derives classical integer oxidation states. To make these methods comparable, we chose to sample whether an atomic charge sign from the *LOBSTER* computations is positive or negative and compare it to the charge signs from the BVA method as implemented in *pymatgen*. For the two approaches to agree, all constituent atoms in the crystal structure after one-to-one mapping must be classified the same way, i.e., as cations or anions. Here we see 96% agreement between the *LOBSTER*’s Mulliken charge analysis results and the BVA method. Deviations can be found in compounds having small electronegativity differences between the constituent atom pairs, i.e., for non-ionic compounds. Figure 9 shows the electronegativity difference between atom pairs for compounds where disagreement between BVA and Mulliken atom classification is observed. We highlight the elements where we encounter disagreement in red. A closer look at this figure reveals that a handful of intermetallic, M–H, M–P, and M–B interactions (involving semimetals) are mismatched. An overview of the involved elements is also given as a heatmap in Fig. 8.

*LobsterPy* can evaluate coordination environments directly based on the electronic structure by taking the ICOHP (a covalent bond strength measure) into account^36,70,71^. The ICOHPs are used to determine the neighboring atoms. In this comparison, we only focus on bonds between cations and anions as determined by the Mulliken charges. Based on the shapes formed by the neighboring atoms, distances to ideal reference polyhedra are then used to determine the closest polyhedra. To validate the coordination environments from *LobsterPy*, we are benchmarking them with purely geometrically determined ones as determined by ChemEnv^70^. In ChemEnv, multiple strategies are available to determine coordination environments. Here we use the SimplestChemEnvStrategy to determine the neighbors, which under the hood, uses a Voronoi partitioning scheme. We set the distance and solid-angle cutoffs to recommended values of 1.4 and 0.3, respectively. To only include cation-anion bonds, we again use the BVA method to determine the ideal oxidation states. Comparing the coordination environments detected for each site, we see an agreement for 79% of the sites. Thus, the coordination environments from our database agree very well with those determined by commonly used geometric algorithms.

### Data exploration and utility

**Fig. 10:**
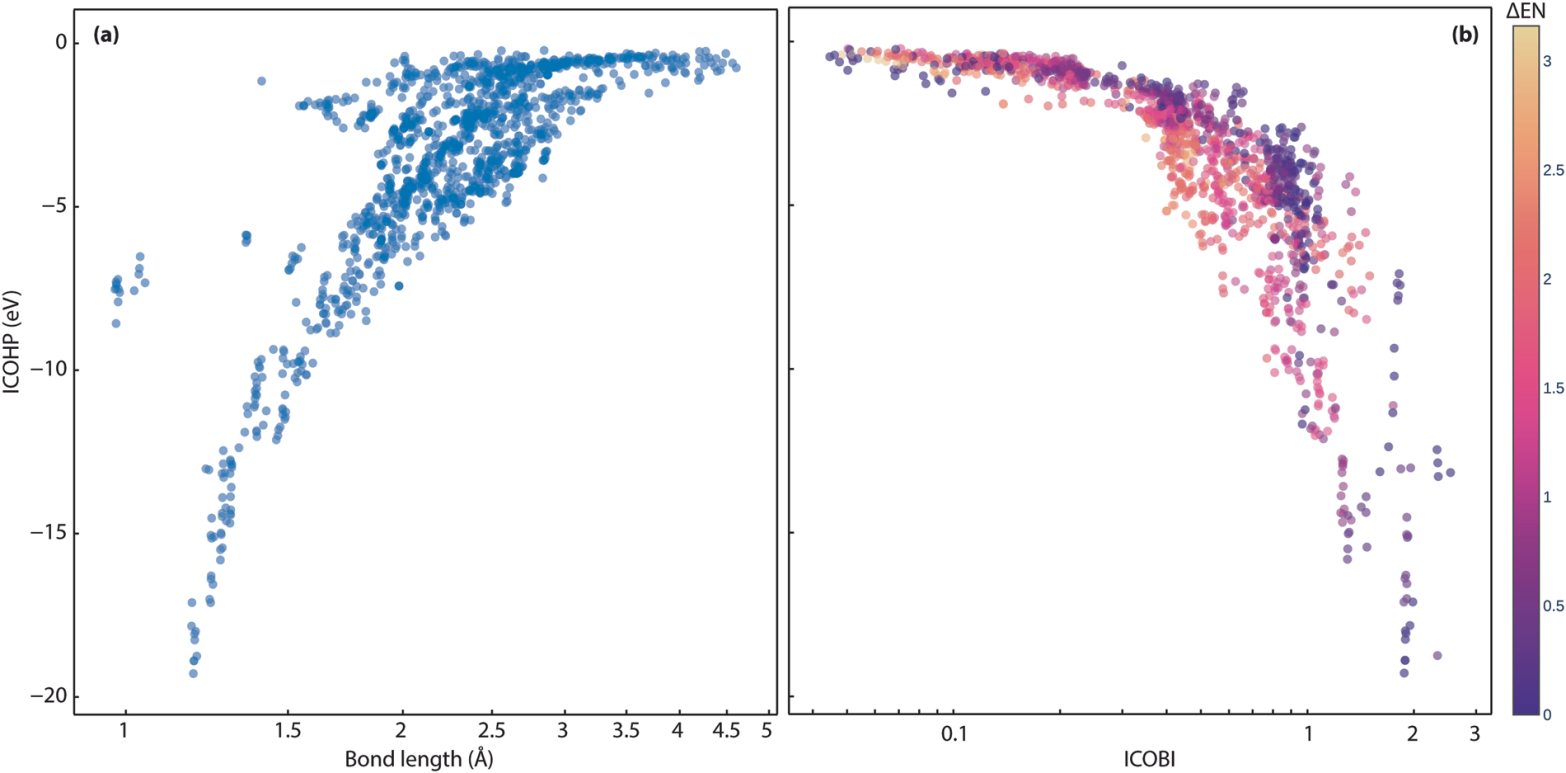
(**a**) The strongest ICOHP values for each compound and their respective bond lengths. (**b**) Strongest ICOHP compared against two-center ICOBI interaction (logarithmic scale). Data points are colored according to the Pauling electronegativity difference between pairs of atoms.

First, we evaluate the bonding indicators in more detail. The most negative ICOHP value indicates the strongest covalent interaction per definition. Plotting the strongest ICOHP values (eV) found per compound and their corresponding bond lengths (Å) as shown in Fig. 10a, we see the expected decrease in covalent bond strengths with increasing bond lengths. In a bond range from about 1 Å to 2 Å, a steep relation between ICOHP and bond distance can be observed, which eventually flattens for longer bond distances, indicating the short-ranged nature of covalency. The outliers around 1 Å within the ICOHP energy range from −5 to −10 eV are O–H and N–H bonds (cf. interactive plots: 10.5281/zenodo.7856484). As covalent bonds between hydrogen and other nonmetal elements are known to be shorter and rather strong in nature^72–74^, this finding is no surprise.

Figure 10b compares the strongest ICOHP and two-center ICOBI interactions for each compound from the *LOBSTER* computations. Each data point is colored according to the Pauling electronegativity difference (ΔEN) between the interacting atoms. More details can be found in the interactive plot (10.5281/zenodo.7856484). Up to a bond order (ICOBI) of 0.3 (weak bond range), the change of the ICOHP with growing ICOBI is smaller than after this value. Then, the covalent bond strength increases rapidly with the bond order, demonstrating the different sensitivity of ICOHP and ICOBI with respect to changes in the chemical bonding environment.

Of course, the more ionic interactions (larger ΔEN) can be found within the smaller ICOHP and ICOBI (absolute) values, as both descriptors indicate covalent interactions until eventually only interactions with small ΔEN dominate for the interval ICOHP < −7 eV and ICOBI > 1. The interactions with very small (absolute) ICOHP and ICOBI values labeled as covalent according to ΔEN are metal-metal (weak covalent) interactions like Rb–Rb or Rb–Cs contacts. Then there is a range of ICOHP (around −0.7 to −2.0 eV) and ICOBI (around 0.25 to 0.45) values containing Zintl-like intermetallic phases like Na_2_TlSb (mp-866132), RbAg_3_Te_2_ (mp-10481), KZnSb (mp-7438), KCuTe (mp-7436), Na_2_AgSb (mp-7392), K_2_AgSb (mp-7643), Na_2_AgAs (mp-8411), K_2_CuSb (mp-10381), K_5_CuSb_2_ (mp-27999), RbTeAu (mp-9008), K_2_SbAu (mp-867335), KAuSe_2_ (mp-29138) or Na_2_AsAu (mp-7773) and more (ΔEN for the respective bonds ranges between 0.1 and 0.5). This is particularly interesting since Zintl phases and related intermetallic compounds are of great interest for thermoelectric candidates^31,75^ and, e.g., Na_2_TlSb^76^ and KCuTe^77^ show thermoelectric behavior. Phase-change and thermoelectric materials contain two-center interactions that tend to show smaller ICOHP and ICOBI values than expected from pure electronegativity differences as they are fragments of (hypervalent) multi-center bonds^4,11,26,62^. In comparison to diamond (ICOHP = −9.6 eV here and in ref. ^4^) and silver (ICOHP = −0.2 eV from ref. ^4^), the two-center bond characteristic regarding the ICOHP lies between metallic and covalent bonding type (such as GeTe with ICOHP = −1.8 eV in ref. ^4^ and ΔEN = 0.09) and is hence related to the metavalent bonding mechanism^26,78–81^. As we have only calculated semiconducting and insulating materials, a purely metallic bonding mechanism can be excluded. Chemically similar compounds in our data set with the classic relation between ICOHP and ΔEN are, e.g., Rb_3_BaS_2_ (mp-9718, ICOHP(As–B) = −7.4 eV, ΔEN = 0.14), BSb (mp-997618, ICOHP = −5.0 eV, ΔEN = 0.01) and Ga_2_Se_3_ (mp-1340, ICOHP = −5.4 eV, ΔEN = 0.74). It needs to be proven if the relevant compounds from our data set exhibit multi-center ICOBI as well, as it would open up a way to use the ICOHP vs. ICOBI plot as a materials map^4,79,80,82,83^ for thermoelectric (and phase-change) materials. In summary, we could demonstrate on a larger scale that ICOHP and ICOBI classify bonds according to covalency, and another indicator would be needed to further distinguish the weak covalent interactions as metallic, ionic, or (potential) multi-center interactions.

**Fig. 11:**
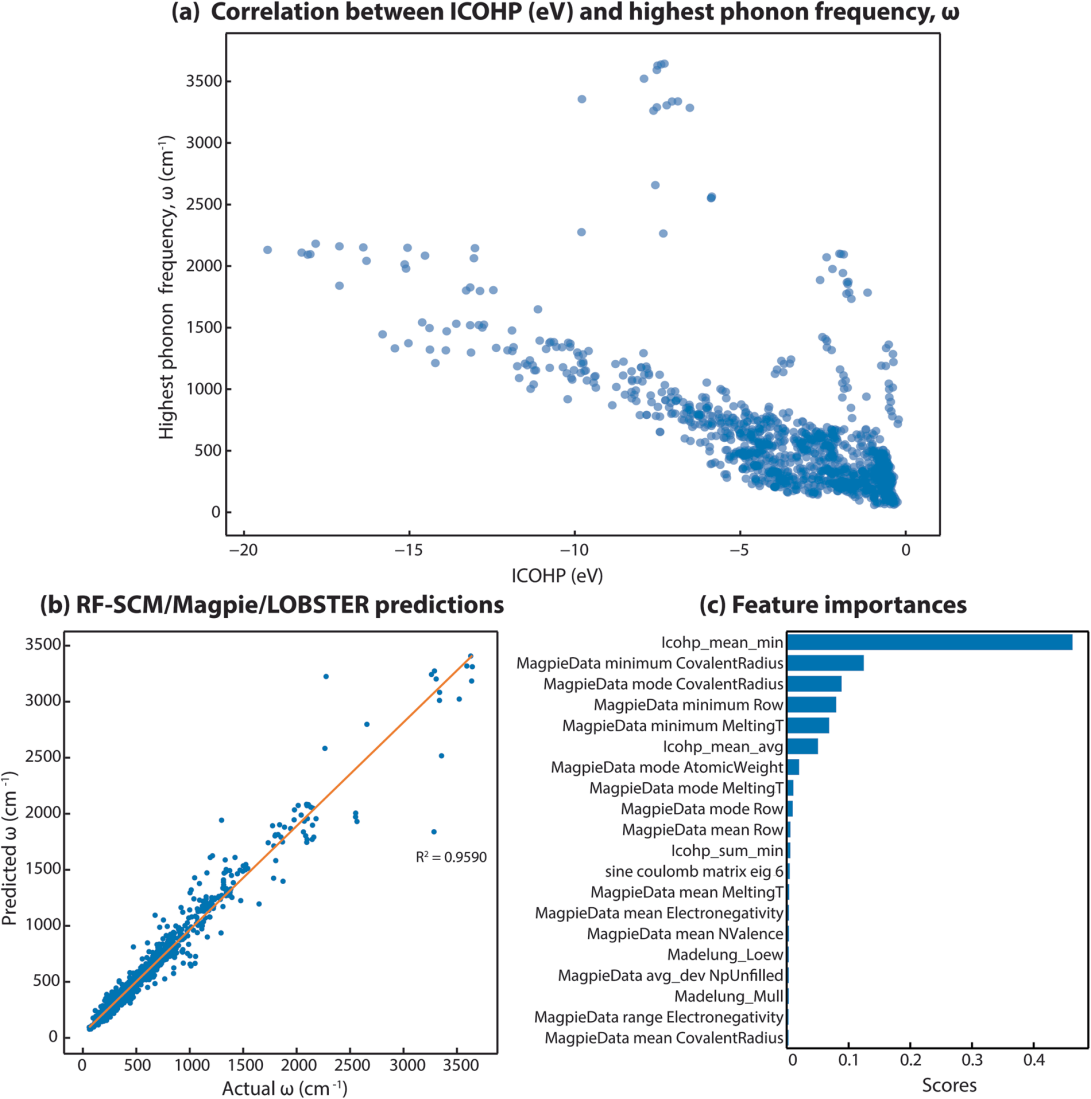
(**a**) The strongest ICOHP (eV) values plotted against the highest phonon frequency, *ω*(*cm*^−1^). (**b**) Predicted *ω* values from RF-SCM/MagPie/LOBSTER model for the whole dataset. (**c**) Feature importance scores for RF-SCM/MagPie/LOBSTER model.

Lastly, we demonstrate the utility of our data by building a machine learning model to predict the highest phonon frequency (*ω*) as computed with harmonic phonon computations^19^. This property is also part of the *MatBench* benchmark set^84^. Therefore, a growing number of ML algorithms, such as *MegNet*^85^, *ALIGNN*^86^, *MODNET*^87,88^ have been used to predict the highest phonon frequency. We selected this property as ICOHP values (covalent bond strengths) have previously been correlated to force constants from harmonic phonon runs (e.g., in ref. ^89^) and should therefore be ideal features for harmonic phonon properties. Also, we have computed *LOBSTER* data for almost all the compounds included in the benchmark phonon dataset in the *MatBench* test suit^84^. We note that bonding analysis only requires a fraction of the computational time of typical phonon runs, as only one static DFT run and post-processing with *LOBSTER* are required. As a first step before developing the ML model, we checked linear correlations between our quantum-chemical bonding information and our target property. We found a clear correlation between the strongest ICOHP of each compound and the highest phonon frequency (*ω*) (Fig. 11a). We can, however, see at least two different trends. We assume this is related to the fact that the highest phonon mode can stem from very different vibrations. Some might be pure stretching vibrations, and others could be collective vibrations involving all atoms. In the first case, mostly one specific bond and one specific ICOHP would have high importance for the phonon mode, whereas in the latter case, all interactions and, therefore, more than one ICOHP within the material would play a role. This observed correlation indicates that using *LOBSTER* data in ML studies as an additional feature could improve the predictive models.

To test this hypothesis, we first transform the data from summarized bonding information (including all types of bonds and not only cation-anion bonds) of the lightweight JSON files to features for our ML models. For this purpose, we developed a featurizer that accepts these JSON files as input and provides mean, min/max, standard deviation of the ICOHP values, and Madelung energies based on Mulliken and Löwdin as an output in a tabular format for each compound. An explanation of the generated features is provided in Table 6.

**Table 6:** ICOHP features extracted using the featurizer for the LOBSTER lightweight JSONs.

Features	Description
Icohp_mean_avg	Average of all relevant ICOHPs per bond at symmetrically inequivalent sites in the structure.
Icohp_mean_max	Maximum of all relevant ICOHPs per bond at symmetrically inequivalent sites in the structure.
Icohp_mean_min	Minimum of all relevant ICOHPs per bond at symmetrically inequivalent sites in the structure.
Icohp_mean_std	Standard deviation of all relevant ICOHPs per bond at symmetrically inequivalent sites in the structure.
Icohp_sum_avg	Average of all relevant ICOHP sums at symmetrically inequivalent sites in the structure.
Icohp_sum_max	Maximum of all relevant ICOHP sum at symmetrically inequivalent sites in the structure.
Icohp_sum_min	Minimum of all relevant ICOHP sums at symmetrically inequivalent sites in the structure.
Icohp_sum_std	Standard deviation of all relevant ICOHP sums at symmetrically inequivalent sites in the structure.
bonding_perc_avg	Average of bonding percentages below the Fermi level from COHPs at symmetrically inequivalent sites in the structure.
bonding_perc_max	Maximum bonding percentage below the Fermi level from COHPs at symmetrically inequivalent sites in the structure.
bonding_perc_min	Minimum bonding percentage below the Fermi level from COHPs at symmetrically inequivalent sites in the structure.
bonding_perc_std	Standard deviation of bonding percentages below the Fermi level from COHPs at symmetrically inequivalent sites in the structure.
antibonding_perc_avg	Average of anti-bonding percentages below the Fermi level from COHPs at symmetrically inequivalent sites in the structure.
antibonding_perc_max	Maximum anti-bonding percentage below the Fermi level from COHPs at symmetrically inequivalent sites in the structure.
antibonding_perc_min	Minimum anti-bonding percentage below the Fermi level from COHPs at symmetrically inequivalent sites in the structure.
antibonding_perc_std	Standard deviation of anti-bonding percentages below Fermi level from COHPs at symmetrically inequivalent sites in the structure.
Madelung_Mull	Madelung energy of the structure derived from Mulliken charges.
Madelung_Loew	Madelung energy of the structure derived from Löwdin charges.

Such an approach is commonly used to generate material descriptors for machine learning of material properties^87,90^. The authors would like to emphasize that the aim of this experiment is not to build the best predictive model but to demonstrate the influence of using *LOBSTER* data as features in ML studies. We assume that graph-based models which allow adding the bonding descriptors as edge features might be more predictive. That being said, to test the influence on a model’s predictive performance, we trained and evaluated two Random Forest (RF) regressor^91^ models. Both models differ only in the input feature sets. RF-SCM/MagPie model consisted of SineCoulombMatrix^92^ and elemental MagPie^90,93^ features (mean, average deviation, range, and max/min statistics) as obtained from the “AutoFeaturizer” module of *Automatminer*^84^ with “debug” preset (180 features). The input feature set and a fixed set of 500 estimators for RF regressor match the Matbench v0.1 RF-SCM/MagPie model^84^. The input feature set of the RF-SCM/MagPie/LOBSTER model consisted of the identical feature space as the RF-SCM/MagPie model, and it was augmented by *LOBSTER* data obtained from our featurizer (199 features). We ensure the train and test sets used for evaluation are identical in both models by setting the same random state seed. The models are evaluated using the nested cross-validation (CV) approach. The inner five-fold CV is used only to optimize the feature selection algorithm (MultiSurfstar^94^) hyperparameter, i.e., the number of features selected. The hyperparameters of the RF regressor are not tuned. The CV statistics across all five test sets for both models are summarized in Table 7.

**Table 7:** Comparison of RF model accuracies across five-fold nested cross-validation test sets.

Model	MAE	Max Errors	RMSE	R^2^
RF-SCM/MagPie	68.047 ( ± 7.502)	1208.329 ( ± 380.017)	149.611 ( ± 19.762)	0.905 ( ± 0.027)
RF-SCM/MagPie/LOBSTER	49.885 ( ± 1.941)	866.373 ( ± 335.674)	100.893 ( ± 9.160)	0.957 ( ± 0.012)

The numbers in the parenthesis depict the standard deviation of the metrics and are given in *cm*^−1^ for MAE, Max Errors, and RMSE. (MAE: Mean absolute error, RMSE: Root mean square errors, R^2^: coefficient of determination).

Our RF-SCM/Magpie model performs similarly to the one reported on the Matbench test suit^84^. Including *LOBSTER* data as features in model input shows an apparent increase in model prediction accuracies. An increase in accuracies by approximately 27% for mean absolute error (MAE), 28% for Max Errors, 32% for root mean squared errors (RMSE), and 5% for *R*^2^ is observed.

On further analysis of the best-performing model (RF-SCM/MagPie/LOBSTER), it is found that the algorithm only needs 50 input features after feature selection for predicting the target values more accurately compared to RF-SCM/MagPie, where all 180 were required. This result demonstrates that significantly fewer features are needed when bonding-related features from *LOBSTER* are included as features. We looked at the feature importance scores readily available for RF models to further analyze the best model. As seen in Fig. 11c, the better performing RF-SCM/MagPie/LOBSTER model shows that the *‘ICOHP_mean_min’* feature, which indicates the ICOHP value for the most covalent bond in a compound, largely contributed to learning the target property of interest. This is the same feature that shows the high correlation in Fig. 11a. Shapley^95^ values computed for the RF models to assess the impact of input features on model prediction also show a similar trend (plots are provided as part of the repository 10.5281/zenodo.7856481). This result further supports our hypothesis that including bonding-related features as material descriptors in ML studies of materials properties not only improves accuracies of predictions but also helps to understand the relationships between material properties and chemical bonding. Here, we clearly see a suspected relationship between covalent bond strengths and harmonic phonon properties.

## Usage Notes

In this work, we provided a Quantum-Chemical Bonding Database to predict and discover new materials. This database consists of summarized COHP-based bonding analysis information ready to be used for ML studies. It also includes (I)COOP, (I)COBI, DOS, atomic charges, and Madelung energies in the computational data JSON files. In addition, we also demonstrated a use-case scenario of how our data could be used for ML studies. This by no means implies that our data should be used in such a manner only. End users are encouraged to explore further.

## Data Availability

The following program versions have been used in this study: *pymatgen* 2022.11.7, *custodian* 2023.3.10, *atomate* 1.0.3, *LOBSTER* 4.1.0, and *VASP* 5.4.4 for *VASP* and *LOBSTER* computations using the workflow. For data validation and processing, we have used *pymatgen* 2023.6.23 and *LobsterPy* 0.2.9. All the scripts used in this study, from starting the workflow, generating data records, reproducing technical validation plots, and ML model evaluations, can be accessed here: https://github.com/naik-aakash/lobster-database-paper-analysis-scripts (10.5281/zenodo.8172527).
